# microRNA‐210 in red blood cells differentially regulates vascular endothelial function between type 1 and type 2 diabetes

**DOI:** 10.14814/phy2.70789

**Published:** 2026-02-23

**Authors:** Tong Jiao, John Tengbom, Eftychia Kontidou, Álvaro Santana‐Garrido, Rawan Humoud, Michael Alvarsson, Kesavan Manickam, Jiangning Yang, Ali Mahdi, Aida Collado, John Pernow, Zhichao Zhou

**Affiliations:** ^1^ Division of Cardiology, Department of Medicine Solna Karolinska Institutet Stockholm Sweden; ^2^ Center for Molecular Medicine Karolinska University Hospital Stockholm Sweden; ^3^ Department of Endocrinology Karolinska University Hospital and Department of Molecular Metabolism and Surgery, Karolinska Institutet Stockholm Sweden; ^4^ Department of Cardiology Karolinska University Hospital Stockholm Sweden

**Keywords:** diabetes, endothelial function, microRNA‐210, oxidative stress, red blood cells

## Abstract

Red blood cells from individuals with type 2 diabetes (T2D RBC) induce endothelial dysfunction due to reduced RBC microRNA‐210 levels, whereas T1D RBCs do not. We hypothesize that microR‐210 plays a protective role explaining this difference. Both male and female adults with T1D and T2D matched for glycated hemoglobin, alongside age‐ and sex‐matched healthy controls, were studied. microR‐210 levels were measured by qPCR. Endothelium‐dependent relaxation (EDR) in isolated rat aortas and nitric oxide (NO) production in endothelial cells following incubation with RBCs were determined using wire myograph and DAF‐FM fluorescence. Protein levels of microR‐210 target PTP1B and the oxidative stress marker 4‐HNE were measured by immunohistochemistry. T1D RBC produced EDR and endothelial NO comparable to healthy controls, whereas T2D RBC impaired both. microR‐210 levels were similar in T1D RBC and healthy controls, but reduced in T2D RBC. microR‐210 inhibition in T1D RBC impaired EDR and increased vascular PTP1B and 4‐HNE, while PTP1B inhibition or mitoTEMPO treatment in aortas improved EDR. RBC microR‐210 regulates endothelial function differently between T1D and T2D by affecting vascular PTP1B and mitochondrial oxidative stress, highlighting a potential therapeutic target to improve vascular health.

## 1 INTRODUCTION

Type 1 (T1D) and type 2 diabetes (T2D), together account for over 95% of all cases of diabetes, are major risk factors for cardiovascular disease through their detrimental impact on vascular function (Joseph et al., 2022). Although sharing a common phenotype of chronic hyperglycemia, there are numerous differences in both types of diabetes in the pathophysiology. T1D is considered an autoimmune disease resulting in insulin deficiency, while T2D is considered to develop due to lifestyle‐associated factors leading to metabolic syndrome such as insulin resistance (Khawandanah, 2019). The function of endothelium, a dynamic regulator of vascular tone and homeostasis, becomes compromised in diabetes due to enhanced oxidative stress and impaired nitric oxide (NO) bioavailability (De Vriese et al., 2000). Thus, endothelial dysfunction is often considered one of the key processes driving the development of cardiovascular disease in both T1D and T2D individuals. However, the cause of endothelial dysfunction remains unclear. Interestingly, increasing evidence highlights that red blood cells (RBCs) undergo dysfunction in several cardiometabolic diseases, contributing to the development of endothelial dysfunction (Kontidou et al., 2023; Tengbom, Humoud, et al., 2024). In fact, RBC dysfunction has been proposed to act as a new mediator of endothelial dysfunction in T2D (Zhou et al., 2018). This effect of RBCs appears to be independent of glycemic status, comorbidity, and medication (Mahdi et al., 2019; Zhou et al., 2018). Existing evidence further reveals that the impact of RBCs on endothelial function was greater in T2D than in T1D, despite matched glycemic levels (Tengbom, Kontidou, et al., 2024). These observations point to distinct mechanisms underlying RBC‐mediated regulation of endothelial function between T1D and T2D, but the exact mechanism driving this difference is unclear.

microRNAs (miRNAs), small RNAs with 19–25 nucleotides long, significantly contribute to transcriptional, post‐transcriptional and post‐translational regulations in cardiometabolic diseases including diabetes (Kontidou et al., 2023). Emerging evidence demonstrates that miRNA‐210 (miR‐210) plays a protective role in cardiovascular homeostasis and acts as a therapeutic target in diabetes‐associated endothelial dysfunction (Collado et al., 2025; Song et al., 2022). Human RBCs serve as a reservoir for miRNAs (Kontidou et al., 2024). They contain abundant miR‐210, whose expression is reduced in individuals with T2D (Kontidou et al., 2024). We further found that such downregulated RBC miR‐210 accounts for endothelial dysfunction (Zhou et al., 2022), indicating that the loss of protective function of miR‐210 in RBCs is a critical mechanism contributing to vascular dysfunction associated with T2D.

Despite these insights, it remains unclear whether RBCs and miR‐210 regulate endothelial function differently in T1D and T2D. Therefore, the present study aims to directly compare RBC‐mediated endothelial function between individuals with T1D and T2D, and to elucidate the role of RBC miR‐210 in the regulation of endothelial function.

## 2 MATERIALS AND METHODS

### 2.1 Human subjects

Eighteen individuals with T1D and 20 with T2D were recruited from the Department of Diabetology and Endocrinology, Karolinska University Hospital and Center for Diabetes, Academic Specialist Center, Health Care Services Stockholm County, Sweden. Individuals matched for fasting glucose (mM: 9.6 ± 3.5 in T1D and 8.6 ± 1.5 in T2D) and glycated hemoglobin (HbA1c) levels (mmol/mol: 64 ± 8 in T1D and 62 ± 13 in T2D). Twenty‐three sex‐matched healthy controls were recruited from a database at the Department of Cardiology, Karolinska University Hospital. All procedures were conducted according to the Declaration of Helsinki and the protocol was approved by the Swedish Ethical Review Authority. Written informed consent was obtained from all participants.

### 2.2 Animals

Male Wistar rats (Charles River) were housed (max. four animals per cage) in the Comparative Medicine facility of Karolinska Institutet and maintained under a 12‐h light/12‐h dark cycles at room temperature, with free access to standard chow (batch no. 24149, SDS diets) and water until 10–15 weeks of age, when experiments were initiated. Rats were anesthetized with pentobarbital sodium (50 mg·kg^−1^, i.p.) and the aorta was removed after thoracotomy. Aortas were cleaned and cut transversely into 2‐mm rings. All animal experiment protocols were approved by the Regional Ethical Review Board of Stockholm and conform to the Guide for the Care and Use of Laboratory Animals published by the U.S.(NIH).

### 2.3 RBC isolation

Whole blood was sampled by puncture of the cubital vein of the participants and collected in EDTA tubes (BD Vacutainer® blood collection tubes; BD Biosciences, San Jose, CA, USA). RBCs were isolated by centrifugation at 4°C with 1000 g for 10 min, followed by three washing cycles with oxygenated Krebs–Henseleit (KH) buffer, as previously described (Zhou et al., 2018). This protocol ensures the removal of 99% of white blood cells and 98% of platelets and results in normoxic oxygen saturation of >99% (Jiao et al., 2022; Yang et al., 2013).

### 2.4 RBC‐vessel co‐incubation and myograph studies

Human RBCs were diluted with KH buffer to hematocrit of ~45% and then were co‐incubated with rat aortic rings in a cell culture incubator at 37°C with 95% O_2_ and 5% CO_2_ for 18 h. After the incubation, the aortic rings were mounted on a wire myograph (Danish Myo Technology A/S) in separate 6‐ml organ baths filled with KH buffer at 37°C and gassed with 95% O_2_/5% CO_2_. Following preconstriction with phenylephrine (10^−6^ M; catalog no. P6126, Sigma‐Aldrich), endothelium‐dependent relaxation (EDR) was evaluated by the administration of cumulatively increasing concentrations of acetylcholine (ACh) (10^−9^ to 10^−5^ M; catalog no. A7000, Sigma‐Aldrich). Endothelium‐independent relaxation (EIR) was assessed by a single concentration of sodium nitroprusside (10^−5^ M; catalog no. S‐0501, Sigma‐Aldrich). To study the function of miR‐210 in the endothelial function, miR‐210 inhibitor, mimic and corresponding scrambled oligonucleotides (50 nM; catalog no. 4464066, 4464058, 4464067, 4464059, Thermo Fisher Scientific) were combined with Lipofectamine RNAiMAX (catalog no. 13778150, Thermo Fisher Scientific), diluted in Opi‐MEM (catalog no. 31985‐062, Gibco), transfected to human RBCs for 18 h and remained in the RBC‐vessel co‐incubation. Hemolysis in the transfected samples was determined by measuring the absorbance of free hemoglobin by optical density at 405 nm with a microplate reader (VANTAstar, BMG LABTECH) (Richards et al., 2015). Free hemoglobin levels of these samples to hemoglobin levels of hemolyzed samples (positive control) were comparable among different groups (mean ± SD, non‐transfection: 0.30 ± 0.09; mimic: 0.24 ± 0.03; scramble: 0.26 ± 0.03). In a separate set of experiments, a cell‐permeable, selective, reversible, and noncompetitive allosteric inhibitor of protein tyrosine phosphatase 1B (PTP1B) (10 μM; CAS no. 765317‐72‐4, Calbiochem Merck): the miR‐210 target (Barile et al., 2014) or the mitochondria targeted antioxidant, mitoTEMPO (100 μM; catalog no. SML0737, Sigma‐Aldrich), was added to the aortic segments in the organ baths for 1 h before preconstriction, followed by the evaluation of EDR to study the possible signaling pathways.

### 2.5 Cell culture

Human carotid arterial endothelial cells (HCtAECs, catalog no. H‐6008; Lonza) were cultured in endothelial cell growth medium (Cell Applications, San Diego, CA), including 10% fetal bovine serum (FBS; Gibco) and 1% penicillin–streptomycin (100 U/mL penicillin and 10 μg/mL streptomycin). The cells were first cultured and expanded in 75cm^2^ flasks (REF no. 83.3911.502, Sarstedt) at 37°C in a humidified incubator with 5% CO_2_ and followed by splitting into 96‐well black microplates (REF no. 94.6120.096, Sarstedt) or 6‐well plates (catalog no. 184959, Thermo Fisher Scientific) until reaching 60%–70% confluence for measuring intracellular NO production and miR‐210 levels.

### 2.6 Determination of intracellular NO production

Intracellular NO was determined using the fluorescent dye DAF‐FM diacetate (catalog no. D‐23841, Thermo Fisher Scientific) following the manufacturer's protocol and previous experience by the authors (Santana‐Garrido et al., 2025). RBCs from individuals with T1D, T2D, and healthy controls were diluted with the growth medium to hematocrit of 1% and incubated with confluent HCtAECs in 96‐well black microplates for 18 h. Following exposure to 10 μM DAF‐FM for 30 min and de‐esterification by incubating with 1× PBS for another 10 min at 37°C, fluorescence was measured in a microplate reader (iMARK, BIO‐RAD), and representative images were taken from a confocal microscope (Nikon ECLIPSE Ti2, Nikon; capture software: NIS‐Elements Viewer) by using 20×/0.8 NA air objective.

### 2.7 qPCR

RNA was extracted from human RBCs and endothelial cells using the Qiagen miRNeasyMini (catalog no. 217004, Qiagen). cDNA and qPCR were performed using TaqMan® Gene Expression Assays (Assay ID: miR‐210, 000512; and U6, 00197, Applied Biosystems) as previously described (Collado et al., 2025; Kontidou et al., 2026). The amplification was performed in a QuantStudio 7 Pro Real‐Time PCR System (Applied Biosystems), and results were normalized to the total mass of the RNA and the U6 as endogenous control. The relative amount of expression was quantified with the 2^−ΔΔCt^ method and presented as fold change. All samples were measured in duplicates.

### 2.8 Immunohistochemistry

Following incubation with transfected RBCs, rat aortic segments were fixed in 4% formaldehyde (24 h), dehydrated, paraffin‐embedded, and mounted on Superfrost® Plus slides. After deparaffinization and antigen retrieval (citrate buffer, pH 6.0), sections underwent peroxidase inactivation (0.3%) and goat serum blockade. Samples were incubated overnight (4°C) with primary antibodies: rabbit monoclonal anti‐PTP1B (1:100 dilution, IgG, catalog no. ab244207, Abcam) and mouse monoclonal anti‐4‐hydroxynonenal (4‐HNE) (1:100 dilution, IgG2b, catalog no. MAB3249, R&D Systems) (Collado et al., 2025). Detection was performed using HRP‐polymer secondary antibodies (REF no. K8000, K8002, K8023, Dako), with isotype controls (Abcam). Isotype controls were used as negative controls (rabbit IgG, catalog no. ab37415 or mouse IgG2b, catalog no. ab18469, both from Abcam). Images were captured with a microscope (Leica DM3000; capture software: LAS V4.3) by using a 40×/0.75 NA air objective. Signals from the entire aortic segment and endothelial layer were analyzed (ImageJ 2.0).

### 2.9 Statistical analysis

Relaxation responses to ACh or SNP were expressed as percentage of preconstriction to phenylephrine. Differences in EDR were analyzed using stacked two‐way ANOVA with mixed‐mode for both treatment and concentration factors and Tukey's multiple comparisons test. Multiple comparisons were performed with ordinary one‐way ANOVA followed by Dunnett's multiple comparisons test or Kruskal–Wallis test followed by Dunn's multiple comparisons test. For two‐group comparison, a paired *t*‐test and Welch's *t*‐test were performed according to pairing and normality tests, including D'Agostino & Pearson test, Anderson‐Darling test, Shapiro–Wilk test, and Kolmogorov–Smirnov test. Fisher's exact test was used for the comparison of categorical data. Data are expressed as mean ± SD, median with interquartile ranges, number or percentage. *p* < 0.05 was considered statistically significant.

## 3 RESULTS

### 3.1 Subject characteristics

Subject characteristics are shown in Table 1. In individuals with T1D, fasting glucose and HbA1c were significantly higher, and total cholesterol and low‐density lipoprotein were lower compared to those in healthy subjects. In individuals with T2D, body mass index, systolic and diastolic blood pressure, fasting glucose, and HbA1c were significantly higher, and total cholesterol and low‐density lipoprotein were lower compared to those in healthy subjects. The individuals with T1D and T2D were slightly older and comprised a higher percentage of smokers compared to healthy controls. Fasting glucose and HbA1c were comparable between individuals with T1D and T2D.

**TABLE 1:** Characteristics of subjects.

Variables	Healthy (*n* = 23)	T1D (*n* = 18)	T2D (*n* = 20)
Age, years	60 ± 6	65 ± 4* ^,^ ^a^	67 ± 7^†††^ ^,^ ^a^
Duration of diabetes, years	NA	34 ± 17	15 ± 5
No. of males	14 (67%)	12 (70%)^c^	14 (70%)^c^
BMI, kg/m^2^	25.3 ± 2.7	28.0 ± 5.0^a^	30.5 ± 5.2^††^ ^,^ ^a^
Systolic BP, mmHg	120 (114–124)	130 (119–134)^b^	133 (128–147)^†††^ ^,^ ^b^
Diastolic BP, mmHg	77 ± 6	74 ± 6^a^	81 ± 6^†^ ^,^ ^a^
No. of smokers	0 (0%)	5 (29%)* ^,^ ^c^	7 (35%)^†^ ^,^ ^c^
Fasting glucose, mM	5.4 ± 0.4	9.6 ± 3.5*** ^,^ ^a^	8.6 ± 1.5^†††^ ^,^ ^a^
HbA1c, mmol/mol	35 ± 2	64 ± 8*** ^,^ ^a^	62 ± 13^†††^ ^,^ ^a^
Hemoglobin, g/L	140 ± 9	139 ± 8^a^	136 ± 14^a^
Creatinine, μmol/L	82 (73–93)	78 (68‐90)^a^	69 (59‐98)^a^
Triglycerides, mmol/L	0.9 (0.7–1.3)	0.9 (0.6–0.9)^b^	1.1 (0.9–1.4)^b^
Total cholesterol, mmol/L	4.9 (4.2–5.9)	3.7 (3.3–4.0)** ^,^ ^b^	3.3 (2.7–3.6)^†††^ ^,^ ^b^
HDL, mmol/L	1.5 ± 0.5	1.4 ± 0.3^a^	1.2 ± 0.3^a^
LDL, mmol/L	2.9 (2.7–3.5)	2.0 (1.4–2.2)** ^,^ ^b^	1.5 (1.3–1.8)^†††^ ^,^ ^b^
Vascular complications, no.			
Coronary artery disease	0	1	4
Retinopathy	0	8	2
Neuropathy	0	6	2
Nephropathy	0	0	1
Peripheral vascular disease	0	1	3
Medication, no.			
ACEi/ARB	0	5	13
Aspirin	0	2	5
Lipid lowering	0	17	15
β‐blocker	0	2	9
Calcium channel i	0	2	9
Insulin	0	17	2
Metformin	0	1	10
GLP‐1 analogue	0	2	11
DPP‐4i	0	0	1
SGLT2i	0	0	9

*Note*: Data are expressed as mean ± SD, *n*, % or median (Q1‐Q3); Welch's *t*‐test was performed to compare the difference of fasting glucose and HbA1c between T1D and T2D.

Abbreviations: ACEi, angiotensin‐converting enzyme inhibitor; ARB, angiotensin receptor blocker; BMI, body mass index; BP, blood pressure; DPP‐4i, dipeptidyl peptidase‐4 inhibitor; GLP‐1, glucagon like peptide‐1; HbA1c, glycated hemoglobin; HDL, high‐density lipoprotein; LDL, low‐density lipoprotein; SGLT2i, sodium‐glucose co‐transporter inhibitor.

^a^ Ordinary one‐way ANOVA followed by Dunnett's multiple comparison test was performed.

^b^ Kruskal–Wallis test followed by Dunn's multiple comparisons test was performed.

^c^ Fisher's exact test was performed.

* *p* < 0.05.

** *p* < 0.01.

*** *p* < 0.001.

^†^ *p* < 0.05.

^††^ *p* < 0.01.

^†††^ *p* < 0.001 versus healthy.

### 3.2 Protective function of RBC miR‐210 is lost in T2D but not in T1D

**FIGURE 1:**
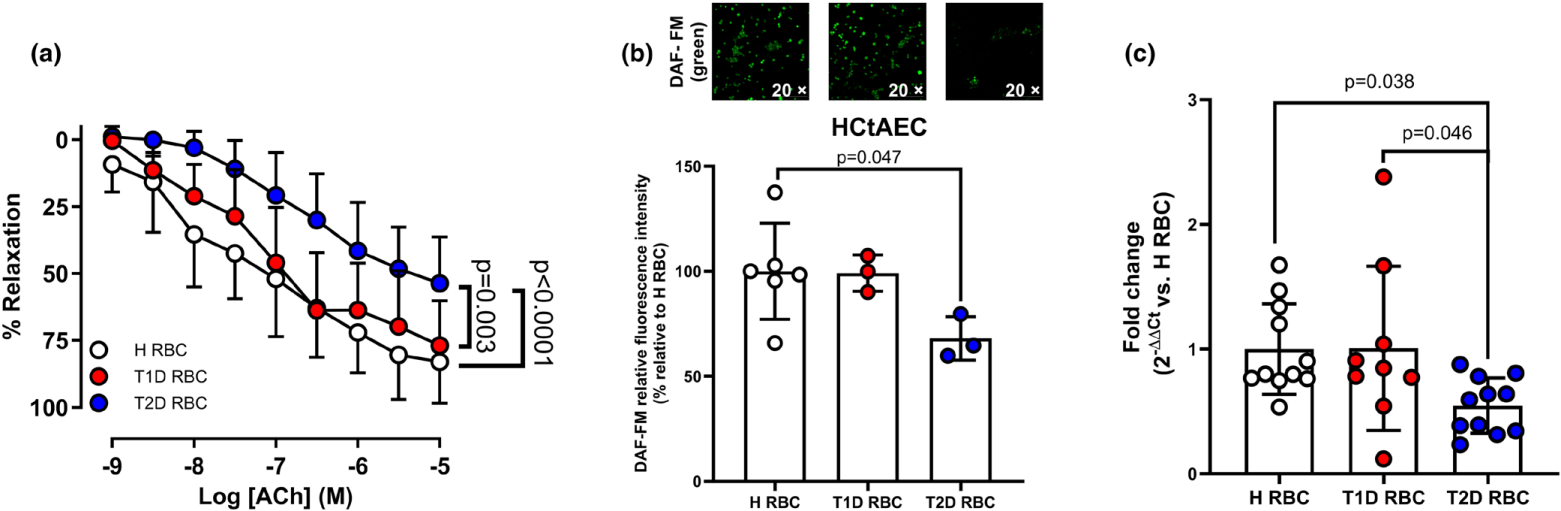
Red blood cells (RBCs) from individuals with type 2 diabetes (T2D) but not T1D induce endothelial dysfunction. (a) Acetylcholine (ACh)‐mediated EDR in aortas following co‐incubation with RBCs from T1D (T1D RBC), T2D (T2D RBC), and healthy controls (HRBC) (*n* = 9–12). (b) DAF‐nitric oxide (NO) fluorescence signal in human carotid artery endothelial cells (HCtAECs) incubated with H RBC, T1D RBC and T2D RBC (*n* = 3–6). Representative images obtained at 20× magnification. (c) miR‐210 expression levels in HRBC, T1D RBC, and T2D RBC (*n* = 9–11). Values are expressed as mean and (or +/−) SD. Two‐way ANOVA and Tukey's multiple comparisons test were performed in a, Kruskal–Wallis test and Dunn's multiple comparisons test were performed in b and ordinary one‐way ANOVA and Dunnett's multiple comparisons test were performed in c.

**FIGURE 2:**
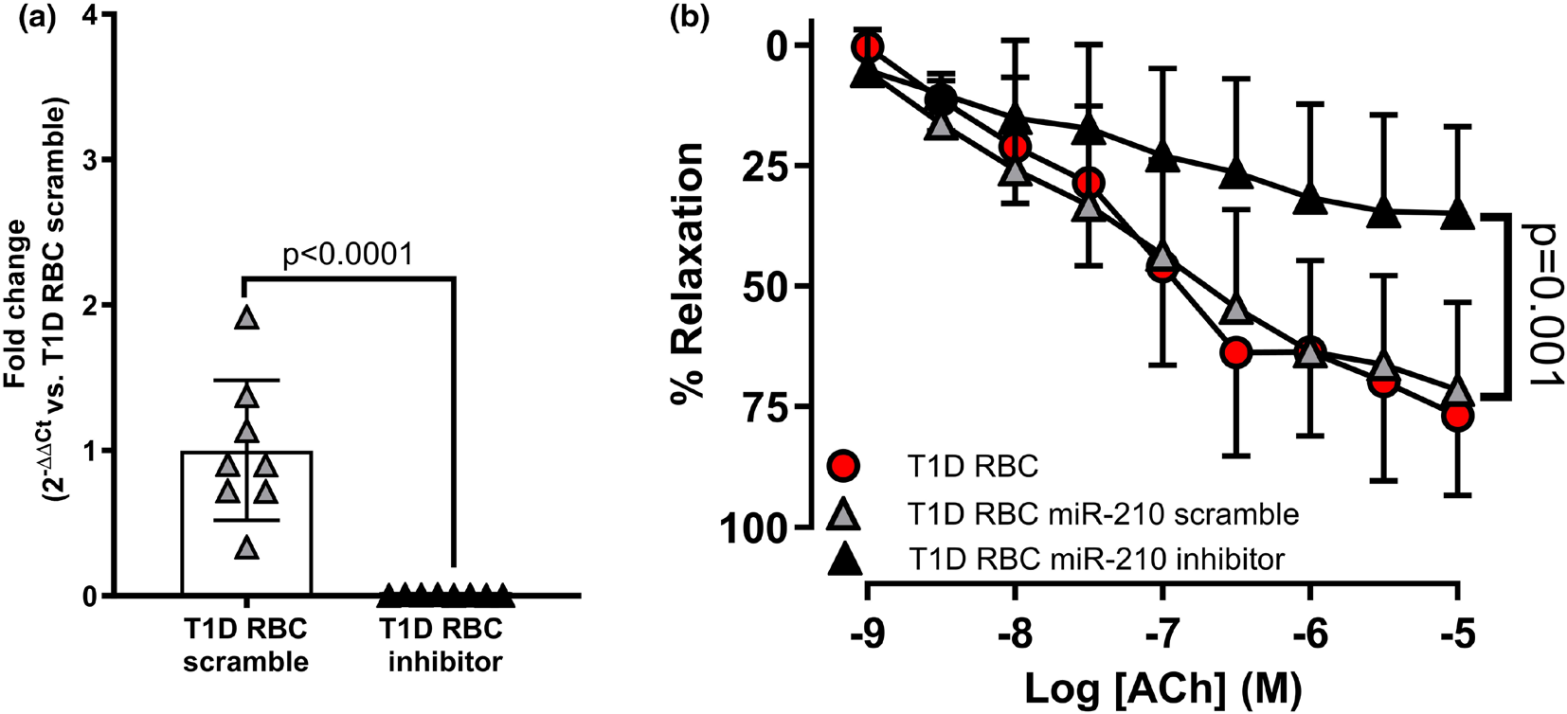
miR‐210 inhibition in RBCs from individuals with T1D induces endothelial dysfunction. (a) miR‐210 expression levels in T1D RBC transfected with miR‐210 scramble or miR‐210 inhibitor (*n* = 8). (b) EDR in aortas following co‐incubation with T1D RBC, T1D RBC transfected with miR‐210 scramble (T1D RBC miR‐210 scramble) or miR‐210 inhibitor (T1D RBC miR‐210 inhibitor; *n* = 8–15). Values are expressed as mean and (or +/−) SD. Paired *t‐*test was performed in a, two‐way ANOVA and Tukey's multiple comparisons test were performed in b.

EDR in aortas incubated with RBCs from T1D was greater than that from T2D, to a level comparable to healthy controls (Figure 1a), despite matched fasting glucose and HbA1cbetween T1D and T2D individuals. EIR was not affected by RBCs from individuals with T1D and T2D, or healthy controls (mean ± SD: 92.4 ± 11.2% vs. 89.2 ± 8.8% vs. 95.3 ± 10.6%, respectively). Similarly, DAF‐FM staining demonstrated that NO production in human endothelial cells remained unaltered after incubation with RBCs from individuals with T1D, whereas NO signal was significantly reduced following exposure to RBCs from T2D patients compared to RBCs from healthy controls (Figure 1b). Based on our previous finding that downregulated RBC miR‐210 contributes to endothelial dysfunction in T2D (Zhou et al., 2022), we determined miR‐210 levels in RBCs in the three groups. We observed that RBC miR‐210 levels were comparably higher in T1D and healthy controls than those from individuals with T2D (Figure 1c). To study the involvement of RBC miR‐210 in regulating endothelial function in T1D, RBCs from individuals with T1D were transfected with either miR‐210 inhibitor or scramble before incubating with rat aortas. miR‐210 expression was significantly reduced following transfection of the miR‐210 inhibitor in RBCs from T1D individuals, which led to significant impairment in EDR compared to groups with the scramble transfection or no transfection (Figure 2a,b). Interestingly, RBCs from individuals with T1D did not affect miR‐210 levels in endothelial cells compared to healthy controls (mean ± SD: 0.980 ± 0.085 in T1D and 1.000 ± 0,190 in controls, respectively. *n* = 3–9). These findings indicate that unlike the endothelial dysfunction induced by downregulated RBC miR‐210 from T2D individuals (Zhou et al., 2022), RBCs from T1D individuals did not damage endothelial function or affect endothelial miR‐210 levels, due to the presence of protective RBC miR‐210.

### 3.3 PTP1B and oxidative stress are involved in RBC miR‐210‐mediated regulation of endothelial function

**FIGURE 3:**
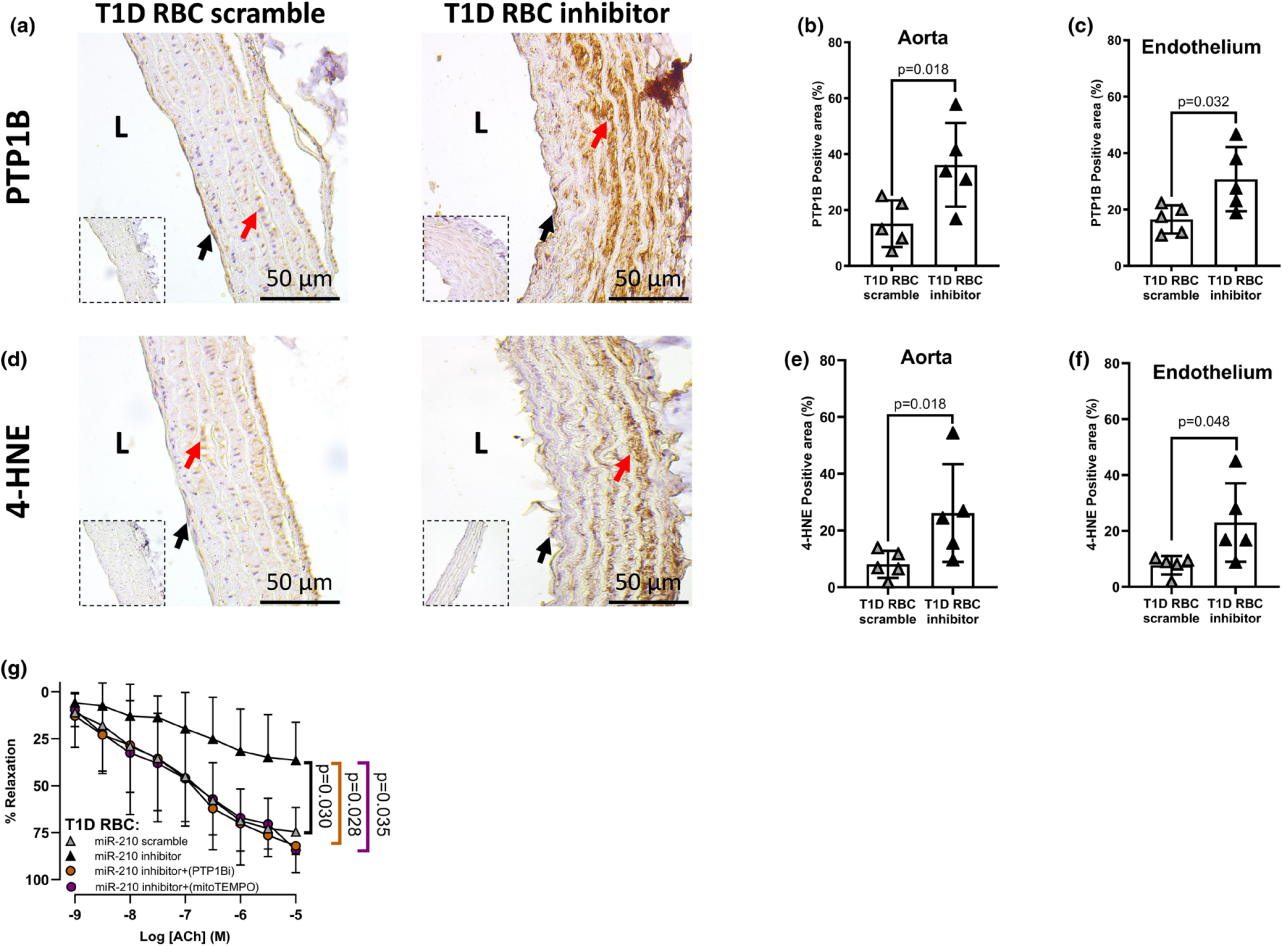
RBC miR‐210 from individuals with T1D suppresses vascular protein tyrosine phosphatase 1B (PTP1B) and mitochondria‐derived oxidative stress. (a–f) Protein expression of PTP1B and 4‐hydroxynonenal (4‐HNE) in aortas incubated with T1D RBC transfected with miR‐210 scramble or miR‐210 inhibitor using immunohistochemistry (*n* = 5). IgG controls are presented in inserts for each experimental condition. L, luminal side of the vessel. Black arrows represent endothelial cells, and red arrows represent smooth muscle cells. (g) Vascular PTP1B inhibition and scavenging of oxidative stress with mitoTEMPO improve EDR in aortas following co‐incubation with T1D RBC transfected with miR‐210 scramble or miR‐210 inhibitor (*n* = 5–7). Values are expressed as mean and (or +/−) SD. Paired *t*‐test was performed in b, c, e, and f. Two‐way ANOVA and Tukey's multiple comparisons test were performed in g.

**FIGURE 4:**
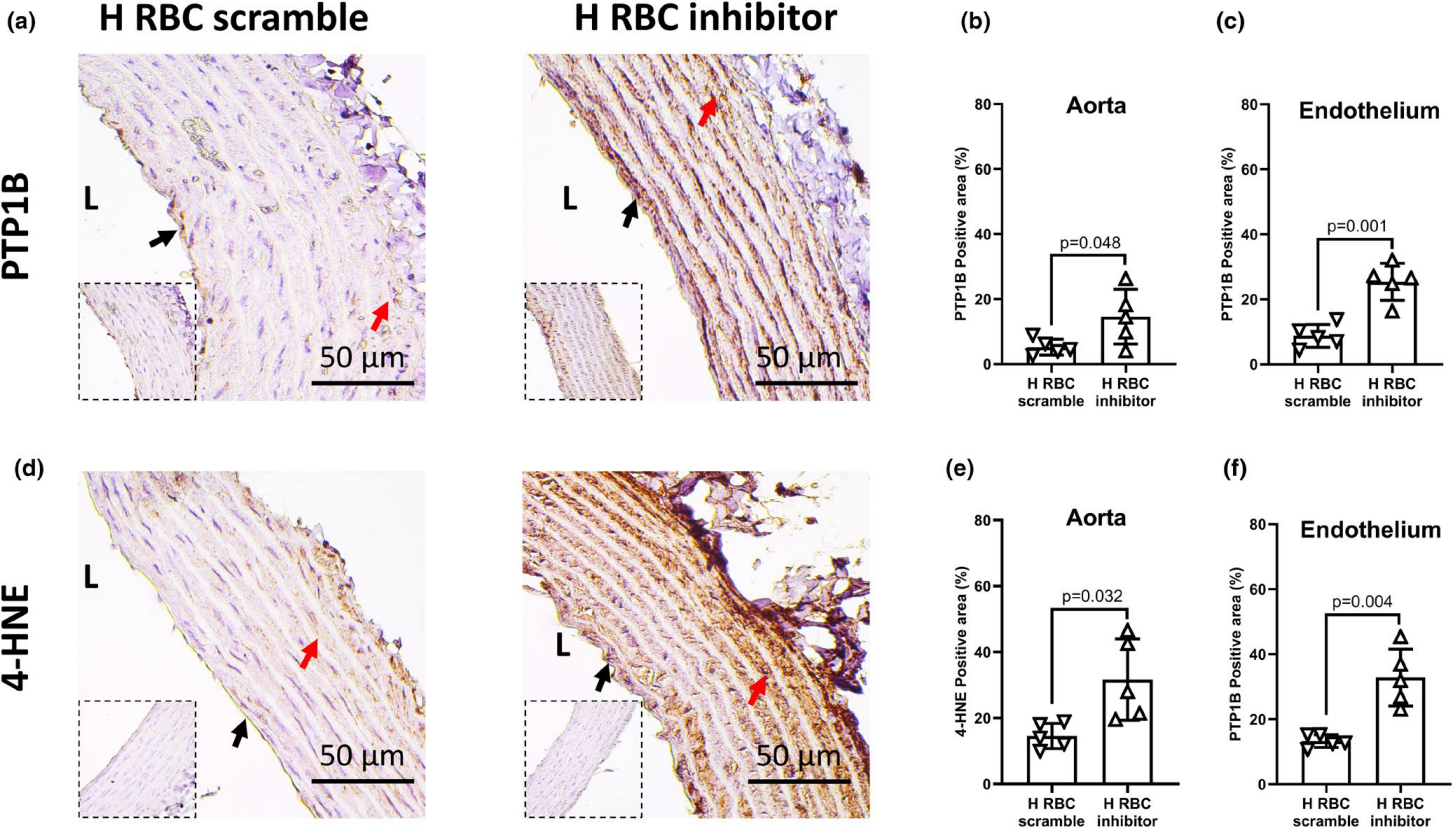
RBC miR‐210 from healthy individuals suppresses vascular protein tyrosine phosphatase 1B (PTP1B) and oxidative stress. (a–f) Protein expression ofPTP1B and 4‐HNE in aortas incubated with RBCs from healthy controls transfected withmiR‐210 scramble (H RBC scramble) or miR‐210 inhibitor (H RBC inhibitor) using immunohistochemistry (*n* = 5). IgG controls are presented in inserts for each experimental condition. L, luminal side of the vessel. Black arrows represent endothelial cells, and red arrows represent smooth muscle cells. Values are expressed as mean +/− SD. Paired *t*‐test was performed in b, c, e, and f.

**FIGURE 5:**
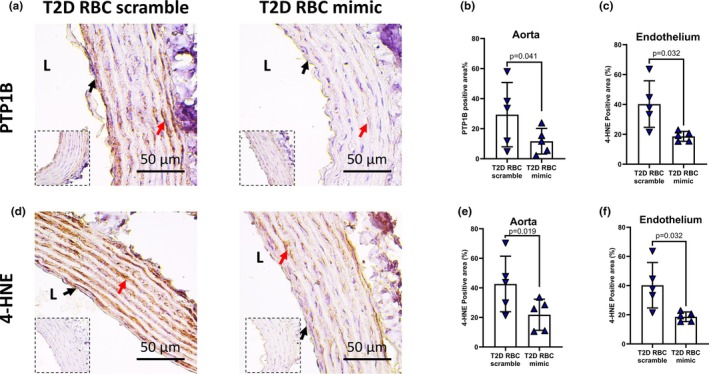
RBC miR‐210 from individuals with T2D promotes vascular protein tyrosine phosphatase 1B (PTP1B) and oxidative stress. (a–f) Protein expression of PTP1B and 4‐HNE in aortas incubated with RBCs from T2D transfected with miR‐210 scramble (T2D RBC scramble) or miR‐210 mimic (T2D RBC mimic) using immunohistochemistry (*n* = 5). IgG controls are presented in inserts for each experimental condition. L, luminal side of the vessel. Black arrows represent endothelial cells, and red arrows represent smooth muscle cells. Paired *t*‐test was performed in b, c, e, and f.

miRNAs exert their function via activating/deactivating downstream targets and miR‐210 has been shown to dysregulate PTP1B and 4‐HNE (Collado et al., 2025). We next studied whether distinct regulation of RBC miR‐210 between T1D and T2D in the control of endothelial function involves miR‐210's downstream target or oxidative stress. Both PTP1B and oxidative stress marker 4‐HNE protein levels were elevated in both endothelium and aortas following incubation with RBCs transfected with the miR‐210 inhibitor from T1D individuals (Figure 3a–f) or healthy controls (Figure 4). In accordance with the previous findings (Zhou et al., 2022), the protein levels of PTP1B and 4‐HNE in both endothelium and aortas were reduced following incubation with RBCs transfected with the miR‐210 mimic from T2D individuals (Figure 5). Of functional importance, inhibition of PTP1B and scavenging of mitochondrial reactive oxidative species (ROS) with mitoTEMPO in isolated aortas attenuated the impairment of EDR induced by RBCs transfected with the miR‐210 inhibitor from individuals with T1D (Figure 3g). These data highlight that distinct regulation of endothelial function by RBC miR‐210 between T1D and T2D involves downstream PTP1B and mitochondria‐derived ROS in the vasculature.

## 4 DISCUSSION

RBCs act as new mediators of endothelial dysfunction contributing to cardiovascular complications in various cardiometabolic diseases including T2D (Kontidou et al., 2023; Tengbom, Humoud, et al., 2024; Tengbom, Kontidou, et al., 2024). We compared the effect of RBCs on endothelial function between individuals with T1D and T2D matched for age and glycemic levels and investigated possible mechanisms involved. We found that (1) RBCs from individuals with T2D but not T1D induce endothelial dysfunction, (2) the protective function of RBC miR‐210 is lost in T2D but not in T1D, and (3) loss of RBC miR‐210 activates downstream vascular PTP1B and oxidative stress in T2D, while maintained RBC miR‐210 suppresses vascular PTP1B and oxidative stress, thereby not damaging endothelial function in T1D. These observations present new insights into differences between T1D and T2D regarding endothelial function regulation and the involvement of RBC miR‐210 as an important mediator.

Deciphering the mechanism behind endothelial dysfunction is essential to uncover the molecular mechanisms that drive vascular complications in diabetic individuals. Our previous study compared endothelial function in T1D and T2D and demonstrated that in vivo endothelial function was markedly impaired in individuals with T2D compared to T1D (Tengbom, Kontidou, et al., 2024). It was suggested that RBCs may act differently in their regulation of endothelial function between T1D and T2D (Tengbom, Kontidou, et al., 2024). Indeed, in the present study using cohorts of subjects matched for age and glycemic status (both fasting glucose levels and HbA1c are comparable between T1D and T2D groups), we show that RBCs do not damage endothelial function in T1D group. The T1D cohort has longer disease duration compared to T2D group. Existing studies demonstrated that disease duration plays a role in endothelial dysfunction in both T1D and T2D patients (Kontidou et al., 2026; Zaharia et al., 2022; Zoungas et al., 2014). Given no detrimental effect of RBCs from T1D patients (with duration of ~34 years) on endothelial function, T1D duration unlikely plays a role in RBC‐mediated endothelial function. Recent evidence highlights a crucial role of T2D duration in RBC‐mediated endothelial dysfunction with RBCs from those with more than 7 years but not less than 1 year since diagnosis damage endothelial function (Kontidou et al., 2026). The present study with all long‐lasting T2D patients further supports this notion. The degree of hyperglycemia is apparently not attributable to the differences in endothelial function or RBC function. This is further supported by the previous study showing that improved glycemic control does not affect RBC‐induced endothelial dysfunction in individuals with T2D (Mahdi et al., 2019). This difference is unlikely to be explained by co‐medications. For instance, all T1D and the majority of T2D individuals were treated with lipid‐lowering medications (mainly statins), which have been shown to protect endothelial function (Margaritis et al., 2014). Several medications previously known to protect endothelial function such as angiotensin converting enzyme inhibitors, metformin, GLP‐1agonists and SGLT2 inhibitors were more frequent in T2D group than in T1D group (Wilcox et al., 2020). However, these medications maybe due to their protective effects have underestimated the difference in RBC‐mediated endothelial dysfunction between the patient groups.

Our findings point to miR‐210 as an alternative mechanism beyond glucose for RBC‐regulated endothelial function between T1D and T2D. Although high glucose can reduce miR‐210 levels and initiate endothelial dysfunction (Collado et al., 2025), the downregulated miR‐210 levels and RBC‐induced endothelial dysfunction persist in individuals with T2D despite improved glycemic control (Collado et al., 2025; Mahdi et al., 2019). In the present study, the RBC miR‐210 levels and RBC‐mediated EDR in aortas and RBC‐produced NO in endothelial cells were unaltered in individuals with T1D, while RBC miR‐210 levels and RBC‐mediated EDR were reduced in T2D, further reinforcing the notion that miR‐210 levels are not regulated by the glycemic level. Insulin resistance, a specific hallmark for T2D, might play a role in our study models and contribute to dysregulation of miR‐210 during erythropoiesis. Although the antioxidant system of glutathione in RBCs seems not to be affected by different degrees of insulin resistance (Galgani et al., 2012), existing evidence shows that miR‐210 levels in whole blood were reduced in obese individuals with insulin resistance compared to those without insulin resistance (Razny et al., 2019). However, the information regarding miR‐210 levels in RBCs is lacking, and the exact role of insulin resistance in the difference in RBC miR‐210‐regulated endothelial function between T1D and T2D warrants further investigations. miR‐210 applies a protective function in several cardiovascular conditions and RBCs contain abundant miR‐210 (Collado et al., 2025; Kontidou et al., 2024; Song et al., 2022). In accordance with the previous observation (Zhou et al., 2022), the protective function of miR‐210 is lost in RBCs from individuals with T2D contributing to endothelial dysfunction. In contrast, miR‐210 expression levels are maintained in RBCs of T1D individuals, which exerts no detrimental effects on endothelial function and may therefore not directly contribute to T1D‐associated vascular complications. Additionally, in contrast to that RBCs reduced endothelial miR‐210 levels in T2D (Zhou et al., 2022), RBCs from individuals with T1D do not affect endothelial miR‐210. The unaltered expression is in line with existing studies where miR‐210 levels in non‐RBC samples from both T1D individuals and animals are not reduced (Assmann et al., 2017; Osipova et al., 2014).

miR‐210 exerts its biological function via regulating downstream PTP1B and mitochondrial ROS in T2D (Collado et al., 2025). In line with a previous study (Zhou et al., 2022), downregulated RBC miR‐210 in T2D promotes downstream PTP1B and oxidative stress in the vasculature. In contrast, maintained miR‐210 levels in RBCs from T1D individuals suppress PTP1B and oxidative stress in the vasculature. This is further evidenced by improved EDR by inhibition of vascular PTP1B and scavenging of mitochondria‐derived ROS following RBC incubation with miR‐210 inhibition. Excessive mitochondrial ROS production induces endothelial dysfunction through multiple cell signaling pathways (Widlansky & Gutterman, 2011). The maintained level of miR‐210 in T1D may inhibit mitochondrial oxygen consumption and reduce mitochondrial ROS flux, therefore maintaining endothelial function. Based on the evidence that both T1D and T2D are significantly associated with cardiovascular dysfunction (Jones et al., 2025) and endogenous levels of PTP1B are upregulated in animal models of both T1D and T2D (Collado et al., 2025; Herren et al., 2015), our observations may suggest a less critical impact of RBCs from T1D individuals on vascular PTP1B signaling and endothelial function.

This study has several limitations. The impact of RBCs from both T1D and T2D on endothelial function was assessed using in vitro and ex vivo models, which may not fully reflect the complexity of cardiovascular condition in vivo. However, these models provide the advantage of isolating the specific contribution of RBCs to vascular function, independent of other circulating cells. A second limitation is that certain medications, including anti‐diabetic drugs, can affect miRNA expression profile (Miao et al., 2025). As discussed above, despite such treatments being unlikely to affect RBC‐mediated endothelial function, their potential impacts on RBC miR‐210 levels are unclear and warrant further investigations.

## 5 CONCLUSION

Our findings demonstrate that the protective function of RBC miR‐210 is lost in T2D but not in T1D, distinctly regulating endothelial function via a mechanism involving downstream PTP1B and oxidative stress. RBC miR‐210‐mediated protective mechanism may provide potential therapeutic strategies for endothelial dysfunction.

## AUTHOR CONTRIBUTIONS

ZZ conceptualized and supervised the study. TJ, JT, EK, ASG, and RH performed and collected research data; TJ and ZZ analyzed research data and performed statistical analysis; MA, AM, and JP recruited patients and collected samples; TJ and ZZ drafted the manuscript; TJ, JT, EK, ASG, RH, MA, KM, JY, AM, AC, JP, and ZZ edited the manuscript. All authors have read and approved the final version of the manuscript.

## FUNDING INFORMATION

This study was supported by the EFSD/Novo Nordisk Foundation Future Leaders Award (NNF22SA0081227 to ZZ), the Swedish Heart and Lung Foundation (20200326, 20220264, 20230386, 20240072, and 20250539 to ZZ; 20220210 and 20250501 to JP; 20241007 to AM; 20250437 to TJ; 20250728 to AC), the Swedish Research Council (2023‐02508 to ZZ and 2024‐02534 to JP), Knut and Alice Wallenberg Foundation (to JP), the Stockholm County Council ALF (FoUI‐972326 to JP; 988725 to AM), the Karolinska Institutet KID grant (202100275 and 202301281 to ZZ), the Karolinska Institutet grant (202201767 and 202402867 to ZZ; 202402554 to TJ; 202402459 to AC), the Swedish Diabetes Foundation (DIA2025‐1028 to ZZ), the Foundation for Geriatric Diseases Karolinska Institutet (2023‐01860 to AC), Tore Nilson's Foundation for Medical Research (2024‐227 to AC), and the Lars Hierta's Memory Foundation (FO2023‐0446 to AC).

## CONFLICT OF INTEREST STATEMENT

No potential conflicts of interest relevant to this article were reported.

## ETHICS STATEMENT

All procedures were conducted according to the Declaration of Helsinki and the protocol was approved by the Swedish Ethical Review Authority. Written informed consent was obtained from all participants.

## Data Availability

Data are available upon reasonable request to the corresponding author.
